# Clinical studies in Myxomatous Mitral Valve Disease dogs: most prescribed ACEI inhibits ACE2 enzyme activity and ARB increases AngII pool in plasma

**DOI:** 10.1038/s41440-025-02109-y

**Published:** 2025-01-21

**Authors:** Smruti K. Nair, Elliot V. Hersh, Kenneth B. Margulies, Henry Daniell

**Affiliations:** 1 Department of Basic and Translational Sciences, School of Dental Medicine, University of Pennsylvania, Philadelphia, PA USA; 2 Department of Oral Surgery and Pharmacology, School of Dental Medicine, University of Pennsylvania, Philadelphia, PA USA; 3 Department of Medicine, University of Pennsylvania, Perelman School of Medicine, Philadelphia, PA USA

**Keywords:** Angiotensin converting enzyme, Angiotensin receptor blocker, inhibitors, Antihypertensive therapies, Plant cell biologics

## Abstract

The hypertension patient population has doubled since 1990, affecting 1.3 billion globally and >75% live in low-and middle-income countries. Angiotensin Converting Enzyme Inhibitors (ACEI) and Angiotensin Receptor Blockers (ARB) are the most prescribed drugs (>160 million times in the US), but mortality increased >30% since 1990s globally. Clinical relevance of Myxomatous Mitral Valve Disease (MMVD) is directly linked to WHO group 2 pulmonary hypertension, with no disease specific therapies. Therefore, MMVD pet dogs with elevated systolic blood pressure treated with ACEI/ARB, were supplemented with oral ACE2 enzyme and Angiotensin1-7 (Ang1-7) bioencapsulated in plant cells. The oral ACE2/Ang1-7 was well tolerated by healthy and MMVD dogs with no adverse events and increased sACE2 activity by 670–755% with ARB (Telmisartan) than with ACEI (Enalapril) background therapy. In vitro rhACE2 activity was inhibited >90% by ACEIs enalapril/benazeprilat at higher doses but lisinopril inhibited at much lower doses. Membrane ACE2 activity evaluated in exosomes was 43-fold higher than the sACE2 and this was also inhibited 211% by ACEI, when compared to ARB. Background ACEI treatment reduced the Ang-II pool by 11-20-fold and proportionately decreased the abundance of Ang1-7 + Ang1-5 peptides. In contrast, ARB treatment increased Ang-II pool 11-20-fold and Ang1-7 + Ang1-5 by 160–260%. Systolic blood pressure was regulated by ARB better than ACEI, despite very high Ang-II levels. This first report on evaluation of metabolic pools in the RAS pathway identifies surprising interactions between ACEI/ARB/ACE2 and significant changes in key molecular dynamics. Affordable biologics developed in plant cells may offer potential new treatment options for hypertension.

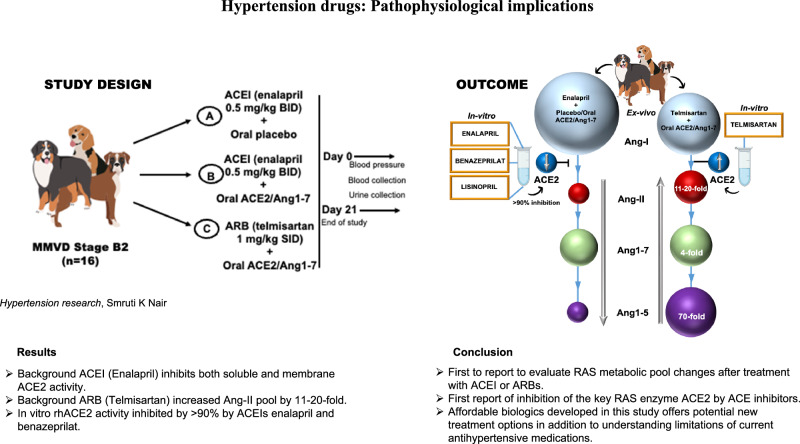

## Introduction

It is estimated that 80% of the population diagnosed with Pulmonary Arterial Hypertension (PAH) live in developing countries with limited access to medical facilities and unaffordable treatment costs [1, 2]. Despite advances in therapy, mortality rate continues to be high with a 7-year survival of only 50% [3]. Patients living with systemic hypertension have doubled since 1990, affecting 1.3 billion people globally and 78% live in low- and middle-income countries. WHO projects that affordable treatments to mitigate hypertension could prevent 76 million deaths by 2050 [4]. Mortality due to hypertension has increased >30% since 1990s and management remains suboptimal in developing countries [5, 6]. Therefore, improved and affordable treatment options are in great need.

ACE inhibitors (ACEIs) and Angiotensin Receptor Blockers (ARBs) are widely used drugs to treat hypertension and associated cardiovascular diseases [7]. Mechanistically, both these drugs reduce the impact of Ang-II pool that causes cardiotoxicity through myocardial remodeling and endothelial dysfunction [8, 9]. ACEIs blocks Ang-II synthesis. ARBs bind to the AT1R and prevent its activation by Ang-II [10]. However, limitations of these drugs include inadequate control of the RAS pathways that regulate blood pressure and the development of compensatory systems when single systems are downregulated [6].

The pro-inflammatory classical arm of RAS - ACE/Ang-II/AT1R is counterbalanced by Angiotensin converting enzyme 2 (ACE2) – a key player of the alternative cardioprotective arm converting Ang-I to Ang1-9 and Ang-II to Ang1-7 [4, 5]. The Ang-II/ATIR complex is central to the pathophysiology of hypertension and progression of cardiovascular disease (CVD) [11]. The downstream metabolites of Ang-I (Ang1-9) and Ang-II (Ang1-7) bind to the Mas and AT2 receptors activating the anti-inflammatory alternative pathway ceasing the cardiotoxic effects of Ang-II [12].

Injectable ACE2/Ang1-7 have been evaluated in the clinic to treat RAS-associated metabolic diseases [13–17] or Covid-19 infection that impacted the RAS pathway [18]. Oral delivery of ACE2/Ang1-7 reduced pulmonary resistance and arterial remodeling in monocrotaline-induced PAH using the rodent model [19, 20]. Here, we evaluate oral ACE2/Ang1-7 in the canine model with Stage B2 Myxomatous mitral valve disease (MMVD) [21]. Although the association between MMVD disease and circulating RAS peptides has been reported [22, 23], the impact of widely used ACEIs/ARBs to treat pet MMVD dogs on the RAS metabolic pools has not yet been investigated. ACEI/ARB drugs are prescribed over 200 million times annually in the United States (https://clincalc.com/DrugStats/Top200Drugs.aspx) and used globally for several decades but there is limited published information on RAS pathway intermediates or enzymes. Therefore, this study investigated the impact of oral ACE2/Ang1-7 in pet MMVD dogs, currently treated with ACEI or ARBs.

## Material and methods

### Ethical statement

The study protocol was reviewed and approved at three clinical sites—The University of Pennsylvania Institutional Animal Care and Use Committee IACUC # 806732, Iowa State University IACUC # 20-186, and University of California at Davis IACUC # 22105. Informed owner’s consents were obtained.

### Myxomatous Mitral Valve Disease dog model

Pulmonary hypertension (PH) is a common complication in dogs with MMVD [24]. MMVD chronically increases pulmonary arterial wedge pressure, causes passive back transmission of increased left-ventricular filling pressure leading to PH [25, 26]. MMVD stage B2 dogs with PH show significantly high levels of circulating Ang-II compared to controls which could also lead to endothelial dysfunction in advanced heart disease dogs [26]. The 16 MMVD dogs enrolled in the study had systolic blood pressure above the normal range (115–138 mm Hg) observed in healthy dogs [22] (Fig. 6D) and an 11–20-fold increase in Ang II was observed in dogs with background ARB treatment. MMVD dogs have systolic pressure in the range of 130–147.5 mmHg [27] similar to the range observed in the current study.

### Blood sample collection

At baseline and study end, blood was collected in Ethylenediaminetetraacetic acid (EDTA) and plain red top tubes for complete blood count (CBC), serum biochemistry, biomarker, and exosome isolation. Serum and plasma for ACE2 activity, Ang1-7, serum RAS peptides, and exosome isolation were stored at −80 °C for batched assays. Samples were analyzed without the addition of protease inhibitors.

### Processing of plasma samples for ACE2 activity assay

Plasma samples were purified by anionic exchange method to remove endogenous inhibitors as previously reported [28, 29]. Briefly, 125 µL of plasma was diluted into 600 µL of low ionic strength buffer (20 mM Tris-HCl, pH 6.5) and incubated for 30 minutes with 100 μL ANX Sepharose 4 Fast-Flow resin at the room temperature on a bench-top rotator. The resin was precipitated for 10 min at 1200 rpm, and then washed twice by removing the supernatant and mixed with 600 µL of low-ionic-strength buffer (20 mmol/L Tris-HCl, pH 6.5) following a centrifuge at 1200 rpm for 10 min. Protein was eluted by supernatant removing and mixed with 250 µL of high salt buffer (1 M NaCl, 20 mM Tris-HCl, pH 6.5) and supernatant-containing protein was decanted following a final spin-down. The resulting eluate (75 µL) was used for ACE2 activity assay for each well in a 96-well plate.

### Preparation of exosomes

The most common technique used to concentrate exosomes is differential centrifugation [30–32]. In the protocol used in the current study, plasma was centrifuged at max speed (5000 rpm; RCF 4000 g) for 10 min at RT in a HERMLE Z326 benchtop centrifuge followed by centrifugation of supernatant at 14,000 rpm (RCF 18,000 g) for 30 min at 4 °C in a Beckman microcentrifuge 22 R, fix angle rotor (F2415P) using 1.5 mL microcentrifuge tubes (PR1MA cat PR-MCT15 MIDSCI). A third ultracentrifugation of the supernatant was done for 70 minutes at 4°C at 35,000 rpm using a Beckman SW 55 Ti swing bucket rotor. Pellets were resolubilized in 1.0 mL of 0.1 μm filtered PBS (Minisart^®^ PES syringe filter code 16,553 K, Sartorious) and centrifuged again in an Optima TLX-120K ultracentrifuge (Beckman) for 90 min at 4 °C at 65,000 rpm using a Beckman TLA100.2 fixed angle rotor (k-factor 12 at maximum speed) in polycarbonate tubes (Beckman catalogue number 343738). Final pellets were resolubilized in 100 μL of 0.1 μm filtered PBS.

### Evaluation of plasma sACE2 and exosome mACE2 activity

ACE2 enzyme activity was measured in both plasma and exosome samples of stage B2 MMVD dogs as described previously with a few modifications [28, 29]. Exosome samples pooled from dogs on ACEI and ARB underwent several cycles of centrifugation that eliminated the endogenous inhibitors and hence did not undergo the purification step. mACE2 activity was evaluated under identical assay conditions as sACE2. Since the samples were processed with EDTA, ZnCl2 concentration was optimized for maximum ACE2 activity (Figures S1, S2). The ACE2 activity was then determined by adding 75 µL of the purified eluate to 25 µL of ACE2 buffer (1.25 M/L ZnCl_2_, 75 mM/L Tris HCl, pH 7.5, and 1 M/L NaCl) followed by incubation with 20 mmol/L fluorogenic Mca-APK(Dnp) ACE2 substrate (R&D Systems, Minneapolis, MN). The enzyme activity was recorded for 1.5 h at 5-min intervals at 28 °C with excitation and emission at 340 nm and 405 nm; with optic position top and gain extended. ΔRFU was calculated by subtracting the 0 min data from the last 90 min data point. sACE2 and mACE2 enzyme activity units were normalized to the protein concentration of the dog plasma sample and reported as pmol/min/mg (mU/mg) = Δpmol/90 min/ mg total protein per well.

### Evaluation of plasma Ang1-7 concentration

Ang1-7 plasma concentration was measured using a competitive ELISA kit (Cloud- Clone Corp., TX, and USA), as described previously with few changes [19, 33]. The resulting Ang1-7 plasma concentration thus estimated was reported in units of pg/mL.

### Evaluation of rhACE2 activity with ACEI/ARB drugs

The ACEI drugs—Enalapril (3, 7, 17, 35, 70, and 140 μg/mL), Benazeprilat (5, 10, 20, 40, and 80 μg/mL), Lisinopril (5, 10, 20, 40, 80, and 160 μg/mL) and ARB drug Telmisartan (5, 10, 20, 40, and 100 μg/mL) were pre-incubated with 0.97 μg/mL rhACE2 (recombinant human angiotensin converting enzyme 2) for 30 min at room temperature (RT). This was followed by the sACE2 enzyme activity assay as discussed above.

### RAS fingerprint analysis

The plasma equilibrium concentration of 10 different RAS APs, including AT1(1-10), AT2(1-8), Ang1-7, angiotensin 1-5 (Ang1-5), angiotensin 2-10 (Ang2-10), angiotensin III (AT3[2–8]), angiotensin IV (AT4[3–8]), and aldosterone, were quantified by liquid chromatography-mass spectrometry/mass spectroscopy (LC-MS/MS) using previously validated and described methods [5, 23, 34].

### Statistical analyses

All sACE2, mACE2 activity, and Ang1-7 assays were performed in triplicates for pooled and duplicates for individual dog samples and data presented as mean ± SEM. For the grouped ACE2 activity and Ang1-7 assay data is presented as mean of individual dogs and confidence interval with a significance of 0.05 (95% CI). A 90–95% CI approach is commonly adopted by the FDA for approval of new drug products [35, 36]. Student’s *t-*test (unpaired) was used for MMVD cohort and control (paired) group comparisons. Correlation analyses were carried out using Pearson correlation. Data from RAS fingerprint analyses are presented from individual dogs with no replicates. Graphs and statistical tests were performed using GraphPad prism 10.0.2 and Microsoft Excel version 16.16.27.

## Results

Based on success of oral delivery of therapeutic proteins in different animal models including rats [19], mice [37], or dogs [38], this study evaluated oral delivery of ACE2/Ang1-7 in MMVD stage B2 dogs. Although previous studies showed delivering oral ACE2/Ang1-7 twice per day had higher efficacy, due to challenges in dealing with pet MMVD dogs at home, only a single oral dose was given in this study while ACEI and ARB were administered twice daily. Safety and short-term efficacy of oral ACE2/Ang1-7 was tested in an initial cohort of 9 MMVD dogs that did not receive other drugs (ACEI, ARB). These data were submitted to FDA for ACE2 gum to neutralize SARS-CoV-2 in the oral cavity and this IND was approved [39]. Subsequent study included long-term efficacy evaluation of oral ACE2/Ang1-7 co-administered with ARB or ACEI in a second cohort of 16 MMVD dogs. Both soluble and membrane-associated ACE2 activity were evaluated in plasma and exosome samples in addition to concentration of plasma Ang1-7. Results observed in MMVD dogs with ARB and ACEI were further verified through in vitro studies using rhACE2. Finally, RAS fingerprint analysis quantified by liquid chromatography-mass spectrometry/mass spectroscopy (LC-MS/MS) revealed different metabolic pools in MMVD groups treated with ACEI, ARB with or without oral delivery of ACE2/Ang1-7.

### Study design

**Fig. 1:**
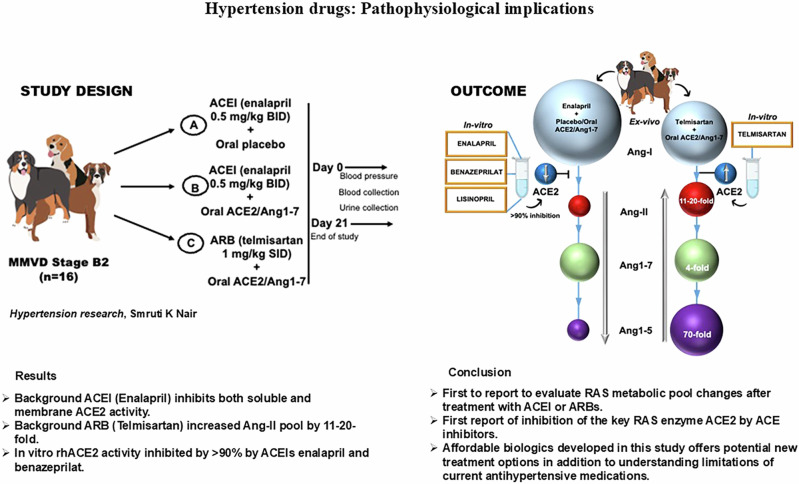
*(Left)* Study design for MMVD stage B2 dogs (*n* = 16) *(Right)* Effect of oral ACE2/Ang1-7 with background ACEI, ARB on sACE2 and mACE2 activity, Ang1-7, Ang1-5 and Ang-II metabolic pools; in-vitro rhACE2 activity inhibition by enalapril/benazeprilat

A cohort of 16 client owned dogs diagnosed with MMVD stage B2 as determined by physical examination and echocardiography were enrolled in a double-blind, modified active-control study (Fig. 1) at three clinical sites (University of Pennsylvania IACUC # 806732, Iowa State IACUC # 20-186 and UC Davis IACUC # 22105). Dogs with body weight >6 kg and age >4 years were included. Additional inclusion criteria included steady therapy of diuretics, (including furosemide, torsemide, thiazides), and pimobendan ~0.25 mg/kg BID within the past 14 days. Pooled plasma samples from 5 healthy dogs (3 F/2 M; mongrel dogs between the ages of 5–10 yrs.) were used as controls for sACE2, mACE2 activity, and Ang1-7 plasma concentration (Table S1). Dogs were excluded based on preexisting renal (sCr ≥2.9 mg/dL) or gastrointestinal disease and medications. Additionally, systolic blood pressure >170 mmHg (evidence of target organ damage), change in blood pressure medications and spironolactone within the past 14 days were excluded. This study design is similar to other studies published in the literature on MMVD dog studies [22, 23, 40].

MMVD stage B2 dogs were randomized to receive either—A (oral plant placebo + ACEI, *n* = 5), B (oral ACE2/Ang1-7 + ACEI, *n* = 5) and C (oral ACE2/Ang1-7 + ARB, *n* = 6) (Fig. 1). The study group design depended on the client’s preference and ongoing treatments. Placebo group was assigned based on availability of MMVD dogs. There were more MMVD dogs treated with ACE inhibitors that enabled ACEI + placebo group but not ARB + placebo group. Dog owners were given daily aliquots of prepackaged lyophilized plant powder (oral ACE2/Ang1-7 and placebo) based on the body weight that they mixed with small amount of canned dog food just before feeding. Treatment included once a day oral administration of plant protein drugs (placebo or ACE2/Ang1-7—1.25/0.6 mg/kg PO qd) in addition to background treatment with either enalapril 0.5 mg/kg BID or telmisartan 1.0 mg/kg SID (Table S2). Dogs who were on other ACEI were switched to enalapril for 14 days until day 0 (Group A and B). In addition, dogs on ACEI were discontinued for 5 days and switched to telmisartan for 14 days until baseline recording on day 0 (Group C). Including the 2-week pre-treatment period, dogs were on ACEI/ARB for 5 weeks till the end of the study period. Clinical chemistry (renal/liver function tests), hematology (complete blood cell counts), NT-pro BNP (a biomarker for advanced cardiac disease) were recorded both before and after the study period on day 21 (Table S3). Dog owners were given daily aliquots of prepackaged lyophilized plant powder based on the body weight that they mixed with small amount of canned dog food just before feeding.

### Oral ACE2/Ang1-7 co-administered with ARB increases both sACE2 and mACE2 activity in MMVD dogs in contrast to inhibition by ACEI

The oral ACE2 used in this study is a full length human ACE2 protein with the transmembrane domain [19]. Since ACE2 is predominantly membrane associated, we compared the impact of oral ACE2/Ang1-7 on enzyme activities of both soluble (sACE2) and membrane-associated ACE2 (mACE2), along with the cleaved product Ang1-7. Plasma samples were pre-treated before measuring the soluble ACE2 (sACE2) activity as described previously [28, 29, 33]. In addition, ZnCl2 concentration was optimized, and we observed maximum ACE2 activity with 0.125 mM (Figure S1) in plasma samples processed with EDTA. Since sonication did not significantly increase the mACE2 activity, further experiments were performed without sonication (Figure S2).

**Fig. 2:**
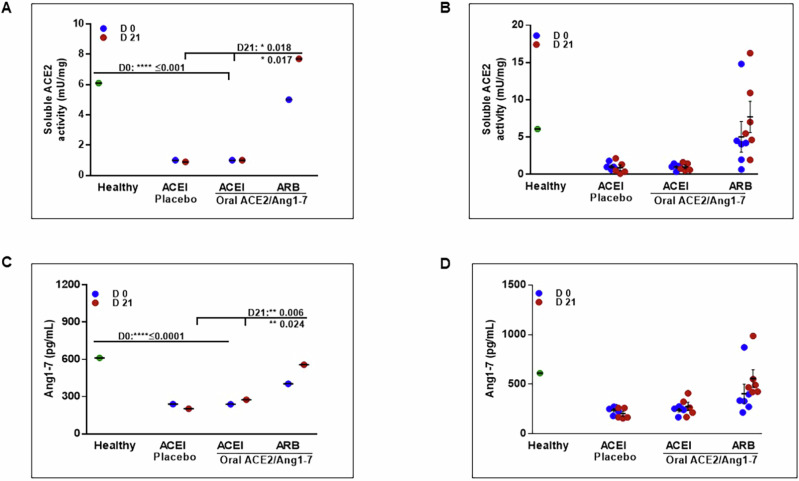
Effect of oral ACE2/Ang1-7 (1.25/0.6 mg/kg PO qD) in ACVIM stage B2 MMVD dogs (*n* = 16) co-administered with Angiotensin converting enzyme inhibitor (ACEI-Enalapril 0.5 mg/kg BID) and angiotensin receptor blocker (ARB-Telmisartan 1.0 mg/kg SID) on plasma soluble ACE2 enzyme (sACE2) activity and Ang1-7 concentration (**A**, **B**) Grouped and individual sACE2 activity on day 0 and day 21. **C**, **D** Grouped and individual Ang1-7 data in MMVD dogs. Healthy pooled sample (green). Grouped data presented as mean (95% CI). Each plasma dog sample was run in triplicates and data presented as mean ± SEM. Pre-study period regime included 2 weeks of enalapril and telmisartan until day 0 and continued for 3 weeks until end of the study

In MMVD dogs maintained on background ACEI or ARB therapy, we assessed the effects of 21-day treatment with plant placebo or oral ACE2/Ang1-7. The sACE2 activity did not statistically differ between MMVD dogs treated with ARB alone and healthy control animals. sACE2 activity in ACEI alone treated dogs averaged one-sixth the level observed in healthy dogs (***p value ≤ 0.001) and did not increase with 21 days of oral plant placebo or oral ACE2/Ang1-7 (Fig. 2A). However, in MMVD dogs with background ARB treatment, baseline sACE2 activity was higher than with background ACEI treatment and increased significantly to 670–755% (*p value = 0.018; *p value =0.017) with 21 days of oral ACE2/Ang1-7 to levels that were higher than those observed in healthy dogs (7.7 vs 6.1 mU/mg) as shown in Fig. 2A, B. Similar results were observed for plasma Ang1-7: reduced levels in MMVD dogs with background ACEI plus plant placebo, persistently reduced levels in MMVD dogs with oral ACE2/Ang1-7 for 21 days, and higher levels with background ARB therapy that was further augmented by treatment with oral ACE2/Ang1-7 for 21 days (**p value = 0.006, *p value = 0.024) (Fig. 2C, D).

**Fig. 3:**
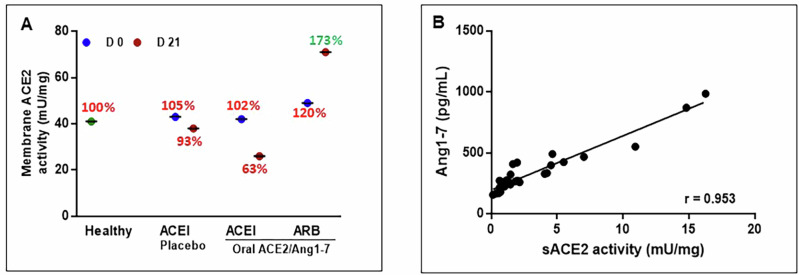
Exosome membrane ACE2 (mACE2) enzyme activity and correlation of sACE2 activity and Ang1-7 concentration in stage B2 MMVD dogs (*n* = 16) co-administered with ACEI-Enalapril (0.5 mg/kg BID) and ARB-Telmisartan (1.0 mg/kg SID) (**A**) Grouped exosome mACE2 activity on day 0 and day 21. The net inhibition % decrease (Red), % increase (green). All pooled samples were run in duplicates and data presented as mean and group comparisons in % change w.r.t the healthy sample. **B** Pearson’s correlation analysis for sACE2 enzyme activity and Ang1-7 in all groups

Contrary to the inhibitory effect of ACEI on sACE2 activity, the mACE2 activity remains similar across ACEI and ARB treated animals with <20% variation compared to healthy controls on day 0 (Fig. 3A). Oral ACE2/Ang1-7 increased mACE2 activity by 73% in the presence of background ARB treatment but inhibited the same by 38% and 12% with background ACEI and oral placebo respectively (Fig. 3A). Sum of mACE2 activity inhibition in oral ACE2/Ang1-7 + ACEI (38%) and increase in oral ACE2/Ang1-7 + ARB (173%) results in 211% of net inhibition by ACEI (Fig. 3A). ACEI significantly reduced (****p value ≤ 0.0001) the Ang1-7 concentration in both oral placebo + ACEI and oral ACE2/Ang1-7 + ACEI by 61% (Fig. 2C). We also observed a very strong positive correlation between sACE2 activity and its product Ang1-7 (*r* = 0.953) (Fig. 3B). These results show the inhibitory effect of ACEI on both sACE2 and mACE2 activity, even in the presence of oral ACE2/Ang 1-7 supplementation that is effective in the presence of background ARB treatment. In addition, ACEI also suppresses the downstream product Ang1-7.

### Comparison of ACE2 activity in exosomes (mACE2) and plasma (sACE2)

Exosomes are small extracellular membrane vesicles that function as a paracrine signaling mechanism packaged by tissues such as kidney and heart [41, 42]. The importance of exosomes as a biomarker for tissue activity has been reviewed and documented [43]. In a rodent animal model with cardiac overload, circulating myocardial exosomes differentially expressed AT2 receptor [44]. In another study with dogs suffering from spontaneous heart disease, exosome miRNA was more specific to disease state and progression compared to circulating miRNA [45]. These studies demonstrate the utility of exosomes as markers of disease and response to treatment modalities by quantifying tissue-level changes. Therefore, in our study, we utilized exosomes to study membrane associated ACE2 (mACE2).

The mACE2 present in the exosomes showed a 7-fold higher enzyme activity compared to sACE2 in healthy dogs. We observe a 10- and a 42-fold increase in mACE2 compared to sACE2 activity with both ARB and ACEI alone treatments respectively (Fig. 2A vs. 3A). On day 21 the sACE2 activity continues to be suppressed by ACEI treatment, confirming inhibition of both endogenous and exogenous ACE2. In the exosome fraction, inhibition of mACE2 activity was observed only after oral delivery of ACE2/Ang1-7. Based on these results we can conclude that exosome profiling of plasma samples helped identify higher mACE2 activity compared to its soluble counterpart. In addition, ACEI drug inhibits sACE2 activity when administered alone and in combination with oral ACE2/Ang1-7 and the mACE2 activity inhibition is evident only after exogenous oral ACE2/Ang1-7.

### Inhibition of rhACE2 activity by ACEI drugs

**Fig. 4:**
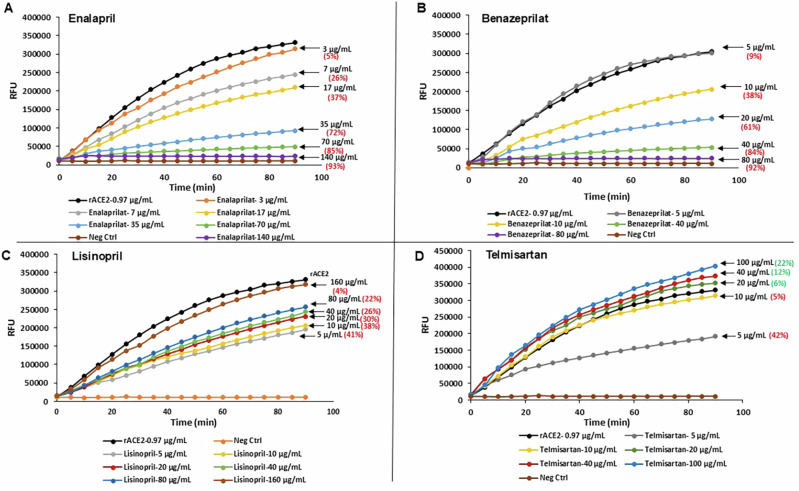
rhACE2 enzyme activity inhibition kinetics of ACEI and ARB drugs. Effect of ACEI drugs—(**A**) Enalapril (**B**) Benazeprilat (**C**) Lisinopril and ARB— (**D**) Telmisartan on rhACE2 enzyme activity

To verify ACEI effect on sACE2 observed in clinical samples, we performed in-vitro ACE2 assays using rhACE2 with ACEI drugs – enalapril, benazeprilat, lisinopril or ARB drug—telmisartan. We observed a dose-dependent inhibition of rhACE2 enzyme activity with both enalapril and benazeprilat. The highest dose of 140 µg/mL enalapril inhibited ACE2 activity by 93%, while 92% inhibition was observed with the highest dose of 80 µg/mL of benazeprilat. Lisinopril showed a different pattern of ACE2 inhibition kinetics (inhibition decreased with drug concentration), with a 40% inhibition with the lowest concentration of 5 µg/mL and 4% inhibition with a higher 160 µg/mL concentration. Although we observed inhibition of the ACE2 enzyme activity at lower ARB telmisartan drug concentrations of 5 and 10 µg/mL, higher concentrations of 20, 40, and 100 µg/mL increased the enzyme activity (Fig. 4A–C). This data reinforces the impact of ACEI/ARB on sACE2 activity in MMVD dogs at doses used in the clinic and hence doses analyzed using rhACE2 are physiologically relevant. Although ACEI and ARB plasma concentrations are reported in human clinical studies [46–49], concentration in urine is several thousand-fold higher than in plasma and no data is available on drug concentration in tissues, where ACE2 is predominantly located.

### Grouped RAS fingerprint analysis of Ang-II, Ang1-7, and Ang1-7 + Ang1-5 pools

**Fig. 5:**
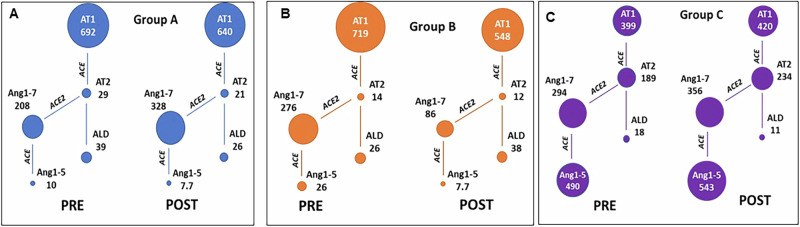
Effect of oral ACE2/Ang1-7 (1.25/0.6 mg/kg PO qD) with background ACEI-Enalapril (0.5 mg/kg BID) and ARB-Telmisartan (1.0 mg/kg SID) and on angiotensin peptide pools using RAS Fingerprint analysis (Grouped data) (**A**–**C**) Spheres indicate the relative concentrations of angiotensin peptides (pmol/L) on day 0 and day 21 after treatment with oral plant placebo + ACEI − group **A** (blue spheres), oral ACE2/Ang1-7 + ACEI − group **B** (Orange spheres) and oral ACE2/Ang1-7 + ARB − group **C** (purple spheres). AT1-Ang-I (1-10); AT2-Ang-II (1-8); ACE-angiotensin converting enzyme, ACE2-angiotensin converting enzyme 2, ALD-aldosterone. One dog (Tater) is excluded from the group C data set

A comprehensive RAS fingerprint analysis of the effect of ACEI/ARB on circulating RAS peptides was performed using liquid chromatography - mass spectrometry [23]. Based on the mechanism of ACEI, we observe a 73% and 80% increase in the substrate Ang-I followed by a 7- and 14-fold reduction in the product Ang-II in the ACEI-reated groups compared to ARB on day 0 (Fig. 5). The Ang1-7 levels were similar among the groups. The Ang1-5 pool decreased by 49- and 19-fold in ACEI alone in oral placebo and oral ACE2/Ang1-7 groups compared to ARB alone group. Compared to MMVD controls, there is a 7- and 3-fold reduction (73 vs 10, 26 pmoL/L) in Ang1-5 pool with ACEI alone [23]. We observe a 6-7-fold increase in the Ang1-5 pool by ARB alone compared to MMVD controls. The uninhibited action of ACE by ARB constantly metabolizes Ang1-7 to Ang1-5. The Ang1-7  + Ang1-5 pool is higher by 260% and 160% with ARB alone as compared to treatment with ACEI alone (Fig. 5). With oral ACE2/Ang1-7 on day 21, ACEI continued to suppress the Ang-II pool by 11-20-fold and the Ang1-7 and Ang1-5 pools reduced by 68%, and 70% compared to ACEI alone. Ang1-5 increased by 70-fold with oral ACE2/Ang1-7 + ARB in comparison with ACEI treatment. (Fig. 5A–C). The Ang1-5 + Ang1-7 pool is reduced by 69% in contrast to 15% increase with ACE2/Ang1-7 + ARB on day 21. In summary, in contrast to ARB, ACEI significantly reduces the Ang-II peptide pool which in turn reduces the abundance of Ang1-7 + Ang1-5 peptides.

### Individual RAS fingerprint analysis of Ang-II, Ang1-5, and Ang1-7 + Ang1-5 pools and systolic blood pressure measurements

**Fig. 6:**
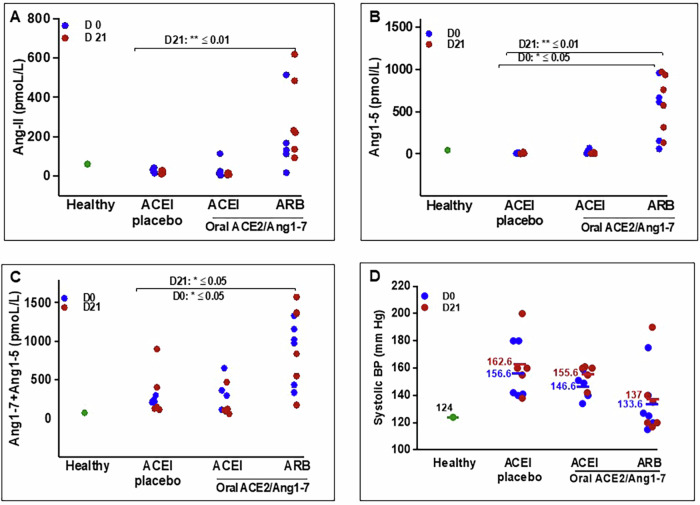
Effect of oral ACE2/Ang1-7 (1.25/0.6 mg/kg PO qD) co-administered with ACEI-Enalapril (0.5 mg/kg BID) and ARB-Telmisartan (1.0 mg/kg SID) on angiotensin peptide pools and blood pressure in stage B2 MMVD (*n* = 16). **A** Ang-II (**B**) Ang1-5 and (**C**) Ang1-7 + Ang1-5 (**D**) Systolic blood pressure measurements. Data represents single measurements and hence no error bars are included. Dogs on other ACEI were switched to enalapril and telmisartan for 2 weeks until the start of the study and continued for 3 weeks of the study period. All dogs were on ACEI/ARB for 5 weeks

The individual dog data presented in Fig. 5A–C confirms the increase in Ang-II pool which subsequently metabolizes to Ang1-7 and Ang1-5 by ARB compared to inhibition of these pools by ACEI as seen in Fig. 4 compared to healthy dogs (data from previously published results) [22] on day 0, the Ang-II, Ang1-5, Ang1-7 + Ang1-5 pools increased by 8-, 11- and 12- folds respectively in dogs treated with ARB alone (Fig. 6A–C). Although MMVD dogs were on ACEI for at least 5 weeks, the systolic blood pressure remained above the normal range (115–138 mm Hg) [22]. Contrary to ACEI, ARB alone on day 0 and with oral ACE2/Ang1-7 regulates BP slightly better within the normal range (day 0–133.6 vs 156.6, 146.6; day 21–137 vs 162.6, 155.6) although not statistically significant (Fig. 6D).

## Discussion

The clinical relevance of MMVD is directly related to WHO Group 2 pulmonary hypertension (PH): pulmonary venous hypertension. Group 2 PH has well-known etiologic links to various types of left heart disease (cardiomyopathy, mitral valve disease, post-procedure pulmonary vein stenosis). Importantly, Group 2 PH has poorly defined molecular mechanisms and no disease-specific therapies. [49]. Pulmonary hypertension (PH) is a common complication in dogs with MMVD [24]. MMVD chronically increases pulmonary arterial wedge pressure, causes passive back transmission of increased left-ventricular filling pressure leading to PH [25, 26]. Our study is also relevant to systemic hypertension. MMVD dogs enrolled in the study had systolic blood pressure above the normal range (115–138 mm Hg) observed in healthy dogs [22] and an 11–20-fold increase in Ang II was observed in dogs with background ARB treatment. Therefore, this study defines previously unappreciated RAAS molecular dynamics and drug responses to alternative, clinically employed RAAS inhibitors in a large animal model with real-world heterogeneity is an important and clinically relevant contribution.

In this study, we evaluated changes in the RAS pathway after oral delivery of ACE2/Ang1-7 bioencapsulated in plant cells, in the setting of either background ACEI or ARB treatment in MMVD dogs. We observed significant ACEI inhibition of both soluble and membrane-associated ACE2 enzyme activity and reduction in the concentration of its product Ang1-7. ACEI also inhibited exogenously delivered ACE2 to MMVD dogs. We further observed that different ACEI agents inhibit recombinant human ACE2 activity to different levels. In contrast, ARB therapy did not inhibit ACE2 activity and therefore an increase in ACE2 activity was observed after oral delivery of ACE2. However, ARB dramatically enhanced the Ang II pool. Potential mechanisms for the differential effects of ACEI vs. ARBs on sACE2 and mACE2 activity include differences in their effects on plasma Ang-II levels (ACEIs directly block Ang-II synthesis whereas ARBs do not) and differences in their ability to directly inhibit ACE2. To the best of our knowledge, this is the first detailed study on RAS metabolic pool on ACEI/ARB treatment. Although ACEI/ARBs are widely prescribed (>200 million annual prescriptions in the US), clinical data on changes in RAS metabolic pathway is very limited, with inconclusive results. [50–52].

The first key finding in the current study is significant ACEI inhibition of both soluble and membrane-associated ACE2 enzyme activity and reduction in the concentration of its product Ang1-7. ACEI was known previously to inhibit ACE enzyme whose product Ang-II has pathological consequences [53, 54]. However, there is no report published on ACEI inhibition of ACE2 activity. ACE2 plays an important role in counterbalancing the vasoconstrictive /proinflammatory effects of Ang-II/AT1R by producing cardioprotective Ang1-7/ Ang1-5. The inhibition of ACE2 by ACEI, reported here for the first time, is not surprising from a biochemical perspective. Both ACE and ACE2 are homologs and have high structural similarity and binding domains [55, 56]. Because ACEI was developed several decades ago and ACE2 enzyme has been studied more recently, comparative studies on their activities haven’t yet been done. Based on our study, inhibition of ACE2 activity - both soluble and membranous forms - and an associated decrease in Ang1-7 by ACEI, could be limiting their cardioprotective effects.

ACEIs inhibited ACE2 activity in a dose-dependent manner except lisinopril behaved differently by showing maximum inhibition at the lowest concentration of 5 μg/mL. Lisinopril has higher ACE selectivity [57] and binds to ACE in a highly ordered and extended conformation [58]. Higher concentration of lisinopril will completely block ACE due to selective binding to ACE but at a higher dose of 160 μg/mL, lisinopril inhibits ACE2 only by 4%. The variations observed between different ACEIs could be due to their inherent selectivity to the ACE2 catalytic domain. Increased concentration of Telmisartan from 10–100 μg/mL blocks ATR1 (Angiotensin 2 receptor) resulting in the upregulation of ACE2.

ACE2 shares 42% sequence identity and 61% similarity in a region surrounding the active site with ACE. A single zinc-binding motif and other amino residues critical for ACE activity are conserved in ACE2. As seen in the ACE2 model, evolutionary relationship exists between ACE2 and ACE in addition to the highly conserved active site in ACE2 [56]. This high degree of sequence conservation also means that the ACE2 domain has a similar secondary and tertiary structure as ACE [55]. Overall, the evidence provided indicates that the ACE2 catalytic mechanism closely resembles that of ACE. The catalytic activity of both ACE and ACE2 share similar chloride sensitivity caused by CL1 and CL2 binding sites in ACE and CL1 site in ACE2. The CL1 site residues Arg186, Trp279, and Arg489 of ACE are equivalent to the ACE2 residues Arg169, Trp271 and Lys481 and are critical to chloride sensitivity [59]. In summary, the inhibition of ACE2 by ACEI observed in the study is due to the structural similarities between ACE2 and ACE.

In the ARB treated group, a significant increase in the Ang-II peptide pool is one of the key observations in our study. A similar increase in Ang-II has been observed in heart failure and chronic kidney disease patients treated with ARB [5, 60, 61]. ARB and ACEI have a profound and distinct impact on the Ang-II: ARB alone increases the Ang-II, and Ang 1-5 peptide pools, while ACEI decreases both. Oral ACE2/Ang1-7 further increases Ang1-5 with background ARB while continued suppression is observed with background ACEI. Reduced Ang-II pool is one of the advantages of ACEI, while lower Ang1-7 + Ang1-5 could reduce the cardioprotective effects of the ACE2/Ang1-7 axis. Ang1-7 is metabolized primarily by the ACE2 enzyme either directly from dissolution of Ang-II or indirectly through Ang-I via Ang1-9 intermediate. However, Ang1-7 can be metabolized directly from Ang-1 through neutral and prolyl endopeptidases (NEP, PREP) or alternatively from Ang-II formed by cleavage of prolyl carboxypeptidase (PRCP) and PREP independent of ACE2 [62]. Hence, we do not observe a dramatic difference in the Ang1-7 peptide pool with ACEI and ARB due to the ACE2 independent compensatory mechanisms at play.

There is compelling evidence that links angiotensinogen and angiotensin-II to hypertension, endothelial dysfunction, cardiac hypertrophy, and renal abnormalities [63]. ARB blocks the binding of Ang-II to AT1R which increases the circulating Ang-II levels which was confirmed by the RAS fingerprint analysis [22]. The difference in the Ang-II pool between ACEI and ARB groups did not affect systolic blood pressure (SBP) significantly. Hypertension and heart failure patients on ACEI and ARB maintain SBP within the range of 124–142- and 135–145-mm Hg respectively [64–66]. Comparatively, in the current study, MMVD dogs on ACEI had a marginally higher SBP compared to the well-controlled ARB-treated dogs.

With ACE2 activity inhibition resulting in reduced cardioprotective metabolites Ang1-7 and Ang1-5, in addition to reduced Ang-II substrate, ACEI impacts both classical and alternate RAS pathways. This could contribute to the comparatively higher BP observed in ACEI-treated dogs. The blockage of ATIR activates the Ang-II/AT2R axis lowering the blood pressure by natriuresis, endothelial relaxation, and vasodilation which is counter-modulatory to the AT1R activation [67]. These compensatory mechanisms helped regulate the blood pressure despite elevated Ang-II levels in ARB treated dogs. However, Ang-II can exert both positive and negative impact depending on the length of exposure, but this short-duration study for three weeks is inadequate to evaluate such changes

A matter of concern is the simultaneous activation of Ang-III and IV pathways in turn activating the AT2 and AT4 receptors by Ang-II with the long-term use of ARB [68]. Through the AT2 receptor, Ang-II mediates the synthesis of matrix metalloproteinases that are associated with atherosclerotic plaque rupture [69]. Activation of AT4 receptor by Ang IV can promote inflammation and expression of other proinflammatory factors [70]. Furthermore, Ang-II fails to counterbalance the effect of bradykinin generating reactive oxygen species leading to NO breakdown [71]. International guidelines for treatment of hypertension and cardiovascular renal protection, recommends ACEI as the first line of treatment, especially for patients with other cardiovascular comorbidities due to the ability of ACEI to prolong life in these patients [72]. In this study, we observed dose-dependent inhibition of rhACE2 activity by different ACEI drugs (Enalapril, Benazeprilat and Lisinopril). Among all the ACEI drugs tested, lisinopril showed the lowest inhibition of the rhACE2 activity. Therefore, future studies could include evaluation of beneficial effects both lower doses of lisinopril in combination with orally delivered ACE2/Ang1-7 to correct dysregulation of the RAS pathway, towards beneficial cardiopulmonary health. Lower oral ACE2/Ang1-7 dose administered in the current study (once instead of BID or 10-fold lower than NOAEL based on toxicology studies) due to challenges of drug administration at home by pet owners. Optimal doses of ACE2/Ang1-7 could be administered in future studies to enhance therapeutic potential.

Despite hundreds of millions of prescriptions of ACEIs/ARBs globally, the prevalence of PAH has doubled with >30% increase in mortality since the 1990s and patients live mostly in developing countries [4–6]. PAH treatment is limited by expense, frequent side effects, short half-life requiring continuous/frequent intravenous delivery and suboptimal survival of only 55% at 3 years [1–3]. Oral delivery of ACE2 in PAH rats attenuated PAH development with decreases in right ventricular (RV) hypertrophy, RV systolic pressure, total pulmonary resistance and pulmonary artery remodeling. Cardiac index increased in a dose-dependent manner [19, 73]. Because we have already completed oral ACE2 toxicology studies and an IND is already approved by FDA for the ACE2 chewing gum [39, 74], this product could soon be evaluated in Phase I/II studies in PAH patients. Hypertension patients will have the first opportunity to treat the root cause of their problem (ACE2 deficiency), along with selection of small molecules with less side effects (Lisinopril versus other ACEI).

The rationale for oral delivery of biologics is to reduce cost and increase patient compliance through noninvasive drug delivery. Plant cell bioencapsulation is ideal for oral delivery because biologics are protected from acids and enzymes in the stomach but are released by gut microbes in the small intestine through lysis of plant cell wall [75]. Recently the FDA approved oral Ara h proteins (Palforizia) in plant cells to treat peanut allergy [76] —Palforzia costs <3% of injectable newly developed biologics [77]. Average development cost of a new injectable biologic drug is $2.5 billion [78] but orally delivered biologic drug launching cost is significantly reduced by elimination of expensive fermenters, purification, and cold chain required for storage and transportation. Therefore, orally delivered biologics are ideal for human and veterinary applications [37, 38, 79, 80]. This is especially relevant to diseases associated with RAS metabolic dysregulation [13–17] or caused by infectious diseases like Covid-19 [81]. Therefore, affordable oral biologic developed in this study offers potential solutions, in addition to understanding limitations of current drugs.

## Supplementary information

Supplemental information
