# Correction: Clinical studies in Myxomatous Mitral Valve Disease dogs: most prescribed ACEI inhibits ACE2 enzyme activity and ARB increases AngII pool in plasma

**DOI:** 10.1038/s41440-025-02157-4

**Published:** 2025-02-14

**Authors:** Smruti K. Nair, Elliot V. Hersh, Kenneth B. Margulies, Henry Daniell

**Affiliations:** 1 Department of Basic and Translational Sciences, School of Dental Medicine, University of Pennsylvania, Philadelphia, PA USA; 2 Department of Oral Surgery and Pharmacology, School of Dental Medicine, University of Pennsylvania, Philadelphia, PA USA; 3 Department of Medicine, University of Pennsylvania, Perelman School of Medicine, Philadelphia, PA USA

Correction to: *Hypertension Research*

10.1038/s41440-025-02109-y, published online 21 January 2025

In this article, the order in which the authors appeared in the author list was incorrectly given as Smruti K. Nair, Henry Daniell, Elliot V. Hersh, Kenneth B. Margulies where it should have been Smruti K. Nair, Elliot V. Hersh, Kenneth B. Margulies, Henry Daniell. The original article has been corrected.

