# Supplementary material for: Clinical studies in Myxomatous Mitral Valve Disease dogs: most prescribed ACEI inhibits ACE2 enzyme activity and ARB increases AngII pool in plasma
Source: Hypertens Res. 2025 Jan 21;48(4):1477–90. doi: 10.1038/s41440-025-02109-y (PMC11972962; doi:10.1038/s41440-025-02109-y)
Supplement: Supplementary file 1 — Supplemental information [file 41440_2025_2109_MOESM1_ESM.docx]

**Table S1** – Demographic data including age, weight, sex, and breed of stage B2 MMVD dogs, n=16.

| **Age (yrs.)** | **Group A**  **Oral plant placebo + ACEI** | **Group B**  **Oral Ace2/Ang1-7 + ACEI** | **Group C**  **OralAce2/Ang1-7 + ARB** |
| --- | --- | --- | --- |
| Number of values | 5 | 5 | 6 |
|  |  |  |  |
| Minimum | 6.00 | 8.00 | 8.00 |
| 25% Percentile | 7.50 | 8.00 | 9.50 |
| Median | 11.0 | 10.0 | 12.5 |
| 75% Percentile | 12.0 | 11.5 | 14.0 |
| Maximum | 13.0 | 12.0 | 14.0 |
| Range | 7.00 | 4.00 | 6.00 |
|  |  |  |  |
| Mean | 10.0 | 9.80 | 11.8 |
| Std. Deviation | 2.65 | 1.79 | 2.56 |
| Std. Error of Mean | 1.18 | 0.800 | 1.05 |
| **Sex** |  |  |  |
| Male | 2 | 4 | 5 |
| Female | 3 | 1 | 1 |
| **Weight (kg)** |  |  |  |
| Minimum | 6.20 | 8.20 | 6.10 |
| 25% Percentile | 6.25 | 8.23 | 6.63 |
| Median | 11.2 | 8.70 | 7.62 |
| 75% Percentile | 15.6 | 11.1 | 10.5 |
| Maximum | 18.4 | 13.3 | 12.8 |
| Range | 12.2 | 5.10 | 6.70 |
| Mean | 11.0 | 9.45 | 8.44 |
| Std. Deviation | 5.08 | 2.17 | 2.45 |
| Std. Error of Mean | 2.27 | 0.969 | 1.00 |
| **Breed** | Cavalier King Charles Spaniel (CKCS) =2; Jack Russell terrier=1, Shetland sheepdog=1, miniature Australian shepherd=1 | Mixed breed=2, Cocker Spaniel=1, Jack Russel terrier=1, Miniature Schnauzer=1 | Mixed breed=2, Dachshund=1, Shih Tzu=1, Yorkshire terrier=1, Miniature Schnauzer=1 |

**Table S2** – Dose of oral plant ACE2/Ang1-7 administered in groups B (n=5) and C (n=6).

| **Powder dose (mg/kg/day)** | **Group B**  **Oral plant ACE2** | **Group C**  **Oral plant ACE2** | **Group B**  **Oral plant Ang1-7** | **Group C**  **Oral plant Ang1-7** |
| --- | --- | --- | --- | --- |
| Number of values | 5 | 6 | 5 | 6 |
|  |  |  |  |  |
| Minimum | 90.1 | 103 | 26.3 | 27.3 |
| 25% Percentile | 90.8 | 104 | 26.5 | 29.9 |
| Median | 96.4 | 111 | 27.0 | 32.7 |
| 75% Percentile | 97.8 | 132 | 28.6 | 36.7 |
| Maximum | 98.6 | 135 | 28.7 | 38.5 |
| Range | 8.50 | 32.8 | 2.34 | 11.2 |
|  |  |  |  |  |
| Mean | 94.7 | 116 | 27.4 | 33.0 |
| Std. Deviation | 3.72 | 14.3 | 1.08 | 4.09 |
| Std. Error of Mean | 1.66 | 5.86 | 0.483 | 1.67 |

**Table S3:** Heart disease biomarker, biochemical blood analysis (hematological, liver and renal function, urine) test measurements in stage B2 MMVD dogs on day 0 (D0) and after 21-days (D21) of treatment. Data expressed as median (IQR).

| **Variables** | **Group A (n=5)** | | | **Group B (n=5)** | | **Group C (n=6)** | |
| --- | --- | --- | --- | --- | --- | --- | --- |
|  | **Plant placebo + ACEI** | | | **Oral ACE2/Ang1-7 + ACEI** | | **Oral ACE2/Ang1-7 + ARB** | |
|  | **D0** | | **D21** | **D0** | **D21** | **D0** | **D21** |
| **Heart disease biomarker** |  | | | | | | |
| N-terminal pro-B-type natriuretic peptide (pmol/L) | 572 (250-1141) | 807 (250-1537) | | 260 (250-819) | 366 (250-609) | 500 (250-2924) | 452 (259-2053) |
| **Complete blood count (CBC)** |  | | | | | | |
| Hemoglobin (13-20 g/dL) | 16 (14.1-17.9) | | 15.2 (14.7-16.1) | 15.3 914-16.8) | 15.3 (13.4-15.9) | 13.9 (11.5-16.5) | 13.7 (11-16.3) |
| White blood cell (10^3/uL) | 7.95 (5.44-20.02) | | 7.86 (3.86-15.4) | 6.46 (4.39-8.14) | 6.05 (4.64-8.28) | 7.23 (4.33-15.67) | 7.86 (4.7-16.63) |
| **Liver/Renal Function tests** |  | | | | | | |
| Glucose (70-136 mg/dL) | 103 (96-115) | | 104 (97-107) | 105 (83-114) | 104 (79-120) | 104 (76-112) | 96 (86-107) |
| Blood urea nitrogen (4-27 mg/dL) | 15 (9-39) | | 16 (8-30) | 14 (12-22) | 17 (10-19) | 20 (13-27) | 21 (14-43) |
| Creatinine (0.5-1.6 mg/dL) | 0.8 (0.7-1.3) | | 0.8 (0.6-1) | 0.8 (0.6-0.8) | 0.7 (0.5-0.8) | 0.9 (0.6-1.6) | 0.8 (0.7-1.5) |
| Albumin (2.7-4.4 g/dL) | 3.6 (3.2-4.1) | | 3.7 (3.2-3.9) | 3.8 (3.4-4.1) | 3.9 (3.2-4) | 3.9 (3.4-4.3) | 3.8 (2.9-4.3) |
| Alanine transaminase (12-118 U/L) | 73 (26-210) | | 56 (19-173) | 36 (31-64) | 34 (27-64) | 58 (29-165) | 84 (5-160) |
| Na (145-153 mmol/L) | 143 (140-147) | | 141 (140-147) | 144 (141-145) | 142 (140-146) | 142 (140-148) | 142 (140-146) |
| K (3.7-5.6 mmol/L) | 4.3 (3.9-4.7) | | 4.7 (4.5-5.4) | 4.3 (4.2-4.9) | 4.3 (4.2-5.4) | 5 (4.2-5.1) | 4.8 (4.4-5.2) |
| **Urine specific gravity** | 1.02 (1-1.02) | | 1.02 (1.02-1.025) | 1.02 (1.01-1.054) | 1.03 (1.01-1.06) | 1.02 (1.015-1.036) | 1.02 (1.015-1.034) |


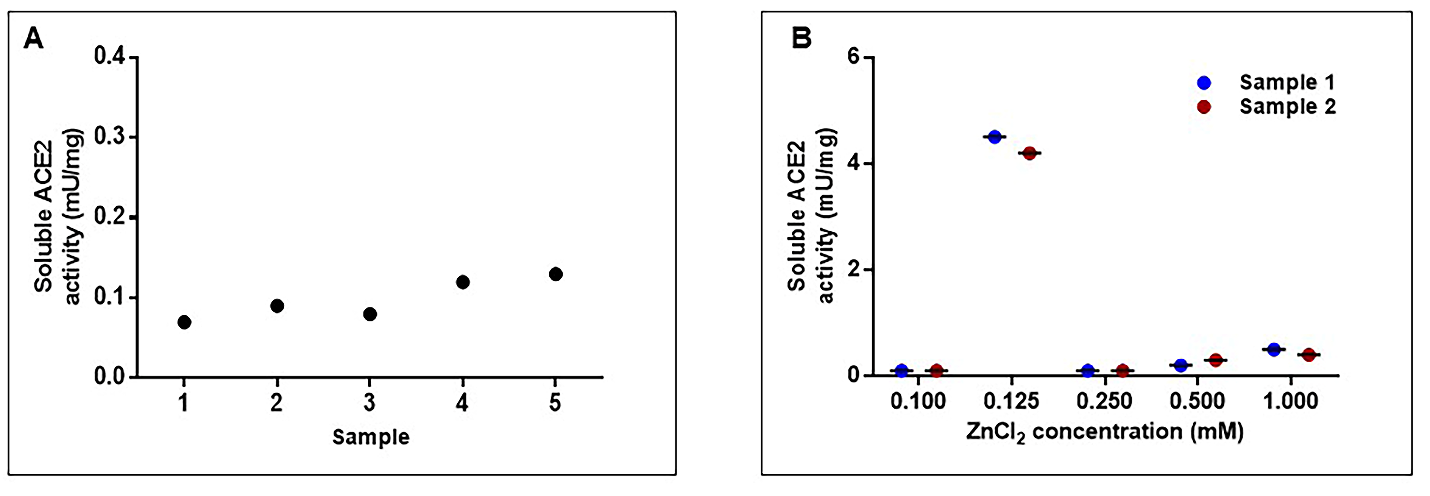


**Figure S1: Optimization of ZnCl_2_ concentration for sACE2 activity measurement in EDTA treated plasma samples (A)** sACE2 activity with 0.1 mM ZnCl_2_ in randomly selected 5 stage B2 MMVD dog plasma samples. **(B)** sACE2 activity measurement after different ZnCl_2_ concentrations of 0.1, 0.125, 0.25, 0.5 and 1 mM were tested in two dog samples revealed maximum recovery of the enzyme activity with 0.125 mM concentration.


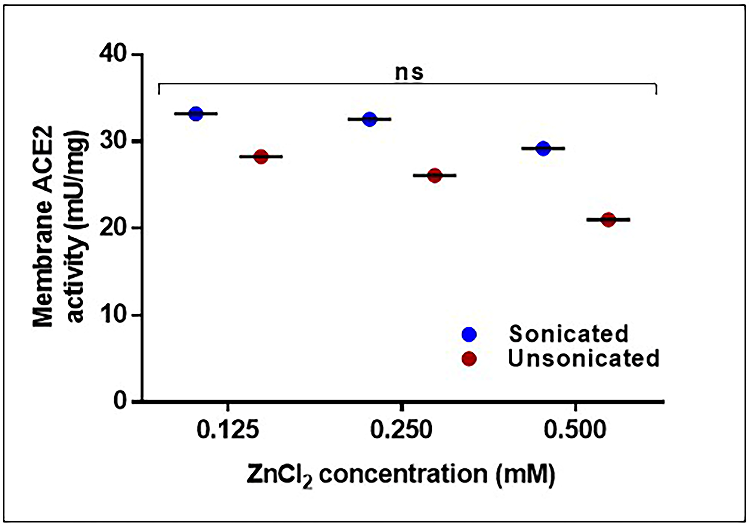


**Figure S2: Optimization of ZnCl_2_ concentration for mACE2 activity measurement in sonicated and unsonicated exosome sample.** mACE2 activity measurement after testing different ZnCl_2_ concentrations of 0.125, 0.25, and 0.5 mM in pooled exosome sample revealed maximum recovery of the enzyme activity with 0.125 mM concentration. No significant difference was observed between sonicated and unsonicated samples.
